# EFFECTS OF SOCIAL RELATIONS ON MORTALITY IN THE CONTEXT OF GRANDPARENTING

**DOI:** 10.1093/geroni/igz038.144

**Published:** 2019-11-08

**Authors:** Heejung Jang, Fengyan Tang

**Affiliations:** University of Pittsburgh, Pittsburgh, Pennsylvania, United States

## Abstract

Issues of health and well-being have received considerable attention as a way to help grandparent caregivers. There is growing evidence that grandparenting is beneficial for grandparent caregivers’ health, yet acting as grandparent caregiver also is detrimental to health and social relations when a grandparent provides an extensive level of care to grandchildren. The extent to which grandparent caregiving benefits or harms of the health of a grandparent is still unknown; mortality specifically has not been systematically studied. Moreover, although altruistic behaviors towards others have been shown to have beneficial effects on caregivers’ health in general, there is little information regarding social relations of grandparent caregivers and their impact on mortality. This study aims to investigate the roles of different aspects of social relations among community-dwelling older adults, examining whether aspects of social relations, including social networks, received functional support aid, and perceived support quality, mediate the association between grandparent caregiving and mortality. The data were drawn from the 2008 and 2014 Health and Retirement Study (N=1,196). Results of survival analyses indicate that custodial and co-parenting grandparents were significantly associated with all-cause mortality over a 6-year period; however, the associations were marginally significant after health statuses were added into the model. Specifically, family-focused network groups were significantly associated with mortality. Received functional support and perceived positive support mediated the association between custodial grandparents and mortality. This study suggests that community-based support may be beneficial to older grandparents and perceived positive relationship quality could matter for older adults’ well-being.

